# Cereblon negatively regulates TLR4 signaling through the attenuation of ubiquitination of TRAF6

**DOI:** 10.1038/cddis.2016.226

**Published:** 2016-07-28

**Authors:** Yoon Min, Sae Mi Wi, Jung-Ah Kang, Taewoo Yang, Chul-Seung Park, Sung-Gyoo Park, Sungkwon Chung, Jae-Hyuck Shim, Eunyoung Chun, Ki-Young Lee

**Affiliations:** 1 Department of Molecular Cell Biology, Samsung Biomedical Research Institute, Sungkyunkwan University School of Medicine, 300, Cheoncheon-dong, Jangan-Gu, Suwon 440-746, Gyeonggi-Do, Republic of Korea; 2 School of Life Sciences, Gwangju Institute of Science and Technology (GIST), Gwangju 500-712, Republic of Korea; 3 Department of Physiology, Samsung Biomedical Research Institute, Sungkyunkwan University School of Medicine, Suwon 440-746, Republic of Korea; 4 Department of Pathology and Laboratory Medicine, Weill Cornell Medical College, New York, NY 10065, USA; 5 Department of Immunology and Infectious Diseases, Harvard School of Public Health, Boston, MA 02115, USA

## Abstract

Cereblon (CRBN) is a substrate receptor protein for the CRL4A E3 ubiquitin ligase complex. In this study, we report on a new regulatory role of CRBN in TLR4 signaling. CRBN overexpression leads to suppression of NF-*κ*B activation and production of pro-inflammatory cytokines including IL-6 and IL-1*β* in response to TLR4 stimulation. Biochemical studies revealed interactions between CRBN and TAK1, and TRAF6 proteins. The interaction between CRBN and TAK1 did not affect the association of the TAB1 and TAB2 proteins, which have pivotal roles in the activation of TAK1, whereas the CRBN-TRAF6 interaction critically affected ubiquitination of TRAF6 and TAB2. Binding mapping results revealed that CRBN interacts with the Zinc finger domain of TRAF6, which contains the ubiquitination site of TRAF6, leading to attenuation of ubiquitination of TRAF6 and TAB2. Functional studies revealed that CRBN-knockdown THP-1 cells show enhanced NF-*κ*B activation and p65- or p50-DNA binding activities, leading to up-regulation of NF-*κ*B-dependent gene expression and increased pro-inflammatory cytokine levels in response to TLR4 stimulation. Furthermore, *Crbn*^*−/−*^ mice exhibit decreased survival in response to LPS challenge, accompanied with marked enhancement of pro-inflammatory cytokines, such as TNF-*α* and IL-6. Taken together, our data demonstrate that CRBN negatively regulates TLR4 signaling via attenuation of TRAF6 and TAB2 ubiquitination.

Cereblon (CRBN) was initially reported as a candidate gene for a mild form of autosomal recessive non-syndromic mental retardation.^1, 2^ Subsequently, different cellular roles of the CRBN protein have been characterized and identified. CRBN interacts with the cytoplasmic region of large-conductance calcium-activated potassium channels, regulating its surface expression.^3^ In the retina, CRBN interacts with voltage-gated chloride channel-2 (ClC-2), thereby influencing assembly or cellular targeting of ClC-2.^4^ CRBN interacts with the *α*1 subunits of AMP-activated protein kinase (AMPK) and inhibits the activation of AMPK.^5^ Importantly, CRBN is involved in thalidomide-induced teratogenicity.^6^ Thalidomide is used in the treatment of leprosy and multiple myeloma.^7, 8^ However, its treatment is associated with limb malformations and other developmental defects.^9, 10^ Although little is known about how these defects are caused, several reports have suggested thalidomide-induced oxidative stress and its antiangiogenic action as a possible cause of teratogenicity.^11, 12^ Recent reports have shown that CRBN forms an E3 ubiquitin ligase complex and functions as a substrate receptor of the E3 ubiquitin ligase complex.^6, 13^ Importantly, CRBN interacts with thalidomide, initiating thalidomide-induced teratogenicity by inhibiting associated ubiquitin ligase activity.^6^ Moreover, it has recently been reported that lenalidomide, a derivative of thalidomide, induces degradation of the lymphoid transcription factors, Ikaros and Aiolos, and casein kinase 1*α* by CRL4^CRBN^ E3 ubiquitin ligase,^14^ indicating that CRBN-binding immune modulatory drugs (IMiDs) differentially regulate CRL4^CRBN^ E3 ubiquitin ligase activity.

So far, many different functions of E3 ubiquitin ligases have been reported.^15, 16, 17^ Several E3 ubiquitin ligases have a crucial role in regulating immune receptor and cellular signaling, and in modulating immune homeostasis and activation.^18, 19, 20^ Among them, tumor necrosis factor (TNF) receptor associated factor 6 (TRAF6), has a pivotal role in innate signaling, including signaling of toll-like receptors (TLRs).^21, 22^ In TLRs-mediated signaling, TRAF6 associates with the dimeric ubiquitin-conjugating enzyme Ubc13/Uev1A and functions as both an adaptor and an E3 ubiquitin ligase-conjugating K63-linked ubiquitin chain attaching to itself and other proteins.^23, 24^ TRAF6 ubiquitination involves the activation of ubiquitin-dependent kinase TAK1, along with binding to TAK1 by several different proteins, such as TAK1-binding protein (TAB)1, TAB2, TAB3, and TAB4.^25, 26, 27^ TAB2 is ubiquitinated by TRAF6, which facilitates assembly of a Toll/interleukin-1 (IL-1) signaling complex containing TRAF6, TAK1, and I*κ*B kinase,^27^ leading to activation of nuclear factor-kappa B (NF-*κ*B) and the production of pro-inflammatory cytokines.^25, 27^

In this study, for the first time, we examine whether CRBN is involved in TLR4-mediated signaling through the regulation of TRAF6 ubiquitin ligase activity. CRBN interacts with the Zinc finger domain of TRAF6, which contains its ubiquitination site, and thereby critically attenuates ubiquitination of TRAF6 and TAB2, leading to inhibition of the production of pro-inflammatory cytokines and NF-*κ*B-dependent gene expression. In line with our *in vitro* results, *Crbn*^*−/−*^ mice exhibited increased mortality, accompanied with marked enhancements of the pro-inflammatory cytokines after challenge with lipopolysaccharide (LPS) *in vivo*, strongly suggesting that CRBN negatively regulates TLR4 signaling through attenuation of TRAF6 and TAB2 ubiquitination.

## Results

### CRBN is negatively implicated in NF-*κ*B activation by TLR4 stimulation

**Figure 1:**
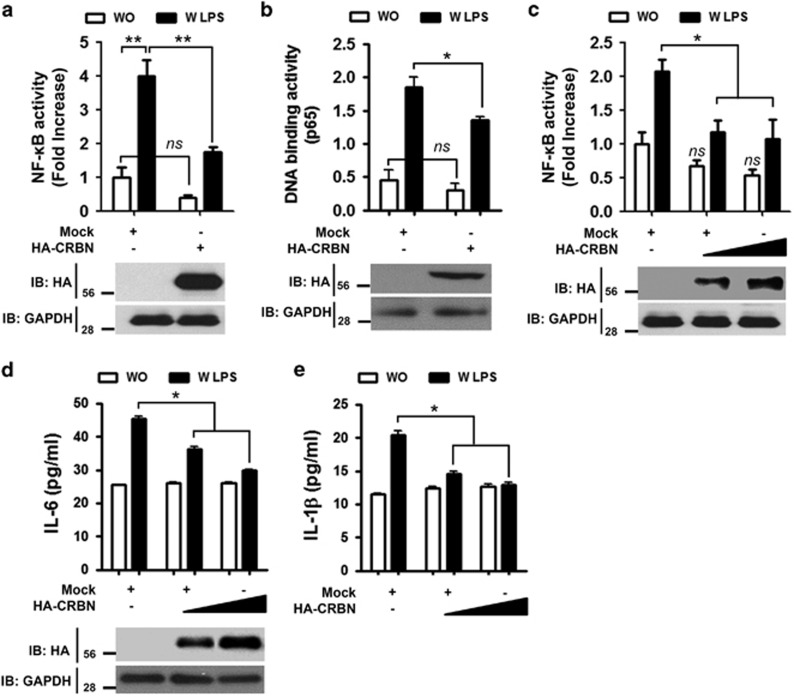
CRBN overexpression inhibits TLR4-induced NF-*κ*B activation. (**a**) 293/TLR4 cells were transfected with a mock or HA-CRBN vector together with a pBIIx-luc and Renilla luciferase vector. Twenty-four hours after transfection, cells were untreated or treated with LPS (200 ng/ml) for 6 h and then analyzed for luciferase activity. Results are expressed as the fold induction in luciferase activity relative to that in untreated cells. All error bars represent standard deviation (S.D.) of the mean from triplicate samples. *ns*, not significant; **P<0.01. (**b**) 293/TLR4 cells were transfected with a mock or HA-CRBN vector, treated with or without LPS (200 ng/ml) for 6 h, and then analyzed for p65-DNA binding activity using the manufacturer's protocol. All error bars represent S.D. of the mean from triplicate samples. *ns*, not significant; *P<0.05. (**c**) THP-1 cells were transfected with mock or different concentrations of HA-CRBN vector together with the pBIIx-luc and Renilla luciferase vectors. Twenty-four hours after transfection, cells were untreated or treated with LPS (200 ng/ml) for 6 h and then analyzed for luciferase activity. Results are expressed as the fold induction in luciferase activity relative to that in untreated cells. All error bars represent S.D. of the mean from triplicate samples. *ns*, not significant; *P<0.05. (**d**,**e**) THP-1 cells were transfected with mock or different concentrations of HA-CRBN vector, treated with or without LPS (200 ng/ml) for 9 h, and production of IL-6 (**d**) and IL-1*β* (**e**) were analyzed by enzyme-linked immunosorbent assay (ELISA). All error bars represent S.D. of the mean from triplicate samples. *P<0.05

To assess a possible role of CRBN in TLR4 signaling for the activation of NF-*κ*B, we transfected 293/TLR4 cells with an NF-*κ*B luciferase reporter in the presence or absence of HA-CRBN. NF-*κ*B reporter activity was markedly increased in mock-transfected cells treated with LPS (Figure 1a, open bar *versus* closed bar in mock), whereas a significant decrease could be seen in CRBN-transfected cells treated with LPS (Figure 1a, closed bar in mock *versus* closed bar in HA-CRBN). In addition, CRBN overexpression significantly inhibited the p65-DNA binding activity induced by LPS stimulation, as compared with mock-transfected cells (Figure 1b, closed bar in mock *versus* closed bar in HA-CRBN). To verify the results observed in 293/TLR4 cells, we transfected THP-1 human monocytic cells with an NF-*κ*B luciferase reporter in the absence or presence of HA-CRBN. Consistently, NF-*κ*B activity induced by LPS stimulation was significantly suppressed by expression of HA-CRBN, as compared to mock-transfected cells (Figure 1c, closed bar in mock *versus* closed bars in HA-CRBN). TLR4 stimulation induces NF-*κ*B activation, leading to the expression of pro-inflammatory cytokines.^22, 28, 29, 30^ Accordingly, we further examined whether CRBN overexpression in THP-1 cells affected the expression of pro-inflammatory cytokines. Upon LPS stimulation in mock-transfected THP-1 cells, IL-6 and IL-1*β* production were greatly enhanced (Figures 1d and e, open bar *versus* closed bar in mock), whereas marked decreases could be detected in HA-CRBN-transfected THP-1 cells (Figures 1d and e, closed bar in mock *versus* closed bars in HA-CRBN), suggesting that CRBN overexpression negatively regulates NF-*κ*B activation by TLR4 stimulation.

**Figure 2:**
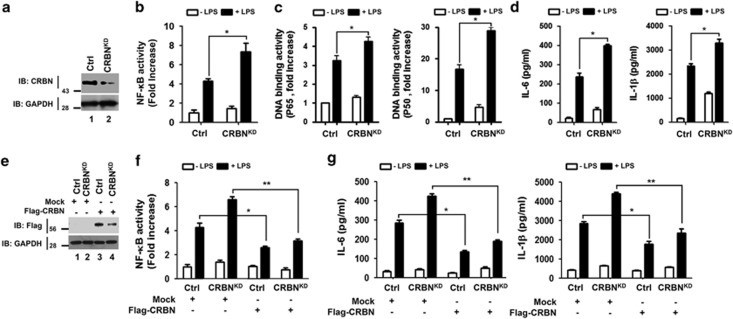
CRBN-knockdown THP-1 cells exhibit increased NF-*κ*B activity and production of pro-inflammatory cytokines. (**a**) THP-1 cells were infected with a lentivirus containing shRNA targeted to human CRBN or control shRNA. Two weeks post-infection, endogenous expression of CRBN proteins were analyzed in CRBN^KD^ and control (Ctrl) THP-1 cells. (**b**) CRBN^KD^ and Ctrl THP-1 cells were transfected with the pBIIx-luc and Renilla luciferase vectors. Twenty-four hours after transfection, cells were untreated or treated with LPS (200 ng/ml) for 6 h and then analyzed for luciferase activity. Results are expressed as the fold induction in luciferase activity relative to that in untreated cells. All error bars represent S.D. of the mean from triplicate samples. *P<0.05 (**c**) CRBN^KD^ and Ctrl THP-1 cells were treated with or without LPS (200 ng/ml) for 6 h and then analyzed for p65- or p50-DNA binding activity using the manufacturer's protocol. All error bars represent S.D. of the mean from triplicate samples. *P<0.05 (**d**) CRBN^KD^ and Ctrl THP-1 cells were treated with or without LPS (200 ng/ml) for 9 h, and production of IL-6 and IL-1*β* were analyzed by ELISA. All error bars represent S.D. of the mean from triplicate samples. *P<0.05. (**e**) CRBN^KD^ and Ctrl THP-1 cells were transfected with a mock or Flag-CRBN vector, as indicated, and then Flag-CRBN expression was examined by western blotting. (**f**) CRBN^KD^ and Ctrl THP-1 cells were transfected with a mock or Flag-CRBN vector (as shown in (**e**)) together with pBIIx-luc and Renilla luciferase vectors. Twenty-four hours after transfection, cells were untreated or treated with LPS (200 ng/ml) for 6 h and then analyzed for luciferase activity. The results are expressed as the fold induction in luciferase activity relative to that in untreated cells. All error bars represent S.D. of the mean from triplicate samples. *P<0.05 and **P<0.01. (**g**) CRBN^KD^ and Ctrl THP-1 cells were transfected with a mock or Flag-CRBN vector (as shown in (**e**)), treated with or without LPS (200 ng/ml) for 9 h, and production of IL-6 and IL-1*β* were analyzed by ELISA. All error bars represent S.D. of the mean from triplicate samples. *P<0.05 and **P<0.01

On the basis of the above results, we verified the functional role of CRBN in CRBN-knockdown (CRBN^KD^) THP-1 cells. To generate CRBN^KD^ cells, THP-1 cells were transduced with a lentivirus containing shRNA targeted to human CRBN or control shRNA sequences. Endogenous expression of CRBN protein was analyzed using a CRBN-specific antibody in both CRBN^KD^ and control (Ctrl) THP-1 cells. CRBN expression was efficiently suppressed in CRBN^KD^ THP-1 cells, but not in Ctrl THP-1 cells (Figure 2a). In contrast, NF-*κ*B reporter activity was significantly higher in CRBN^KD^ THP-1 cells treated with LPS than in Ctrl THP-1 cells (Figure 2b). Furthermore, p65- or p50-DNA binding activity was also enhanced in CRBN^KD^ THP-1 cells compared with Ctrl THP-1 cells (Figure 2c, p65 and p50). Consistently, the production of pro-inflammatory cytokines was significantly higher in CRBN^KD^ THP-1 cells treated with LPS than in Ctrl THP-1 cells (Figure 2d, IL-6 and IL-1*β*). To verify the function of CRBN, we transfected Ctrl and CRBN^KD^ THP-1 cells with mock or Flag-tagged-CRBN (Figure 2e), and performed NF-*κ*B reporter assay. The overexpression of Flag-CRBN in both Ctrl and CRBN^KD^ THP-1 cells led to significant inhibition of NF-*κ*B reporter activity, compared to mock-transfected cells (Figure 2f). Moreover, IL-6 and IL-1*β* production were also significantly attenuated in both Ctrl and CRBN^KD^ THP-1 cells overexpressed by Flag-CRBN, as compared with mock-transfected cells (Figure 2g, IL-6 and IL-1*β*). These results strongly suggest that CRBN negatively regulates TLR4-mediated signaling, thereby inhibits the activation of NF-*κ*B.

### CRBN interacts with TAK1, but has no effect on associations of TAB1 and TAB2 with TAK1

**Figure 3:**
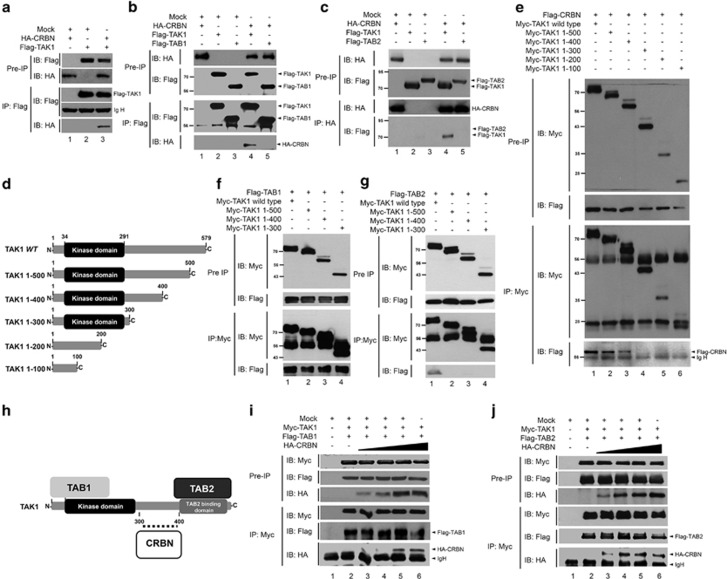
CRBN interacts with TAK1, but not with TAB1 or TAB2. (**a**) HEK293T cells were transfected with mock, HA-CRBN, or Flag-TAK1, as indicated. At 38 h after transfection, transfected cells were extracted, immunoprecipitated with the anti-Flag antibody, and immunoblotting was performed with the anti-Flag or anti-HA antibodies (**b**,**c**) HEK293T cells were transfected with mock, HA-CRBN, Flag-TAK1, Flag-TAB1, or Flag-TAB2, as indicated in each figure. At 38 h after transfection, transfected cells were extracted, immunoprecipitated with anti-Flag (**b**) or anti-HA (**c**) antibodies, and immunoblotting was performed with anti-Flag or anti-HA antibodies. (**d**) TAK1 wild type (wt) and TAK1-truncated mutants: TAK1 1-500, TAK1 1-400, TAK1 1-300, TAK1 1-200, and TAK1 1-100. (**e**) HEK293T cells were transfected with Myc-TAK1 wt or Myc-TAK1 1-500, Myc-TAK1 1-400, Myc-TAK1 1-300, Myc-TAK1 1-200, or Myc-TAK1 1-100, along with Flag-CRBN. At 38 h after transfection, transfected cells were extracted, immunoprecipitated with anti-Myc, and then immunoblotting was performed with anti-Flag or anti-Myc antibodies. (**f**,**g**) HEK293T cells were transfected with Myc-TAK1 wt or Myc-TAK1 1-500, Myc-TAK1 1-400, Myc-TAK1 1-300, Flag-TAB1 (**f**), or Flag-TAB2 (**g**), as indicated in each figure. At 38 h after transfection, transfected cells were extracted, immunoprecipitated with anti-Myc antibody, and then immunoblotting assay was performed with anti-Flag or anti-Myc antibodies. (**h**) A schematic model of the interaction between TAK1 and TAB1, TAB2, or CRBN. (**i**,**j**) HEK293T cells were transfected with Myc-TAK1 and Flag-TAB1 (**i**) or Myc-TAK1 and Flag-TAB2 (**j**) in the presence or absence of different concentrations of HA-CRBN. At 38 h after transfection, transfected cells were extracted, immunoprecipitated with an anti-Myc antibody, and then immunoblotting was performed with anti-Flag, anti-HA, or anti-Myc antibodies

Next, we investigated the molecular mechanism by which CRBN negatively regulates TLR4 signaling. In TLR4 signaling, ubiquitin modification has emerged as an important mechanism that regulates cell signaling for the NF-*κ*B activation.^31, 32^ TRAF6, an important E3 ubiquitin protein ligase, has a key role in the TLR4 signaling pathway. Ubiquitinated TRAF6 has been implicated in the activation of TAK1 through the ubiquitination of TAB2, eventually leading to activation of NF-*κ*B.^23^ Therefore, we assumed that CRBN could be implicated with ubiquitin-mediated activation of TLR4 signaling. Coimmunoprecipitation was used to assess the association of CRBN with various components associated with TRAF6 in TLR4 signaling, including TAK1, TAB1, and TAB2. Flag-TAK1 significantly immunoprecipitated with HA-CRBN (Figure 3a, lane 3). In contrast, HA-CRBN did not coprecipitate with Flag-TAB1 or Flag-TAB2 (lane 5 in Figures 3b and c, respectively). A consistent interaction between CRBN and TAK1 was evident (lane 4 of Figures 3b and c, respectively), suggesting that CRBN interacts with TAK1, but not TAB1 or TAB2.

We further examined whether the interaction of CRBN and TAK1 could affect the association of TAB1 and TAB2 to TAK1. To do this, we determined the TAK1 interaction sites of CRBN, TAB1, and TAB2 using TAK1 truncation mutants (Figure 3d). Flag-CRBN significantly co-precipitated with Myc-TAK1 wt, Myc-TAK1 1–500, and Myc-TAK1 1–400 (Figure 3e), but not Myc-TAK1 1-200 and Myc-TAK1 1-300, suggesting that CRBN interacts with the TAK1 300–400 domain. Flag-TAB1 was significantly co-precipitated with Myc-TAK1 wt and Myc-TAK1-truncated mutants (Figure 3f). In contrast, Flag-TAB2 co-precipitated with only Myc-TAK1 wt (Figure 3g). These results suggest that CRBN, TAB1, and TAB2 may interact with different regions of TAK1 (Figure 3h). To directly confirm the result, Myc-TAK1 and Flag-TAB1 or Flag-TAB2 were co-transfected into HEK293T cells with different concentrations of HA-CRBN. Flag-TAB1 and Flag-TAB2 significantly co-precipitated with Myc-TAK1 in the absence or presence of different concentrations of HA-CRBN (Figures 3i and j, lanes 2 and 3–6, respectively). Moreover, the interaction between TAK1 and CRBN was significantly increased in a dose-dependent manner (Figures 3i and j, lanes 3–6, IB: HA). These results demonstrate that CRBN interacts with TAK1, and that the interaction does not affect the association of TAB1 and TAB2 with TAK1.

### CRBN interacts with TRAF6, which attenuates TRAF6 and TAB2 ubiquitination

**Figure 4:**
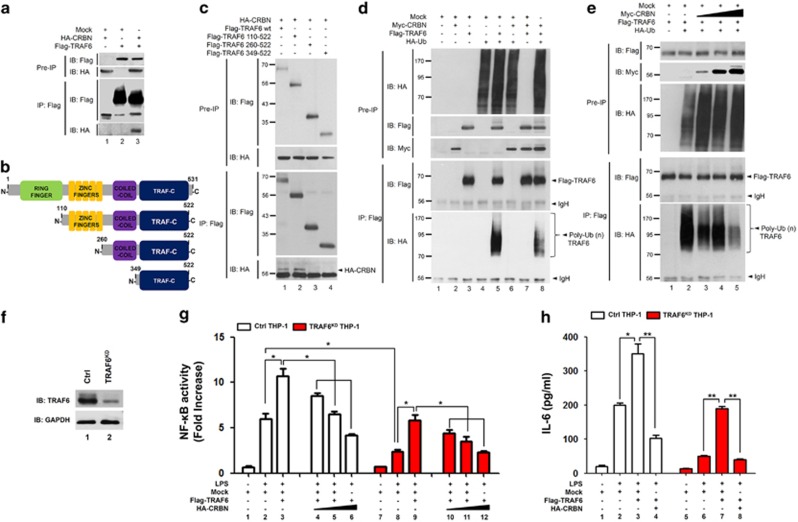
CRBN interacts with TRAF6 and inhibits the ubiquitination of TRAF6. (**a**) HEK293T cells were transfected with mock, HA-CRBN, or Flag-TRAF6, as indicated. At 38 h after transfection, transfected cells were extracted, immunoprecipitated with anti-Flag antibody, and then immunoblotting was performed with anti-Flag or anti-HA antibodies. (**b**) A schematic presentation of TRAF6 wt and its truncated mutants used in this study: TRAF6 110-522, TRAF6 260-522, and TRAF6 349-522. (**c**) HEK293T cells were transfected with HA-CRBN, Flag-TRAF6 wt, Flag-TRAF6 110-522, Flag-TRAF6 260-522, or Flag-TRAF6 349-522, as indicated. At 38 h after transfection, transfected cells were extracted, immunoprecipitated with an anti-Flag antibody, and then immunoblotting was performed with anti-Flag or anti-HA antibodies. (**d**) HEK293T cells were transfected with mock, Myc-CRBN, Flag-TRAF6, or HA-Ub, as indicated. At 38 h after transfection, transfected cells were extracted, immunoprecipitated with an anti-Flag antibody, and then immunoblotting was performed with anti-Flag, anti-Myc, or anti-HA antibodies. (**e**) HEK293T cells were transfected with mock, HA-Ub, and Flag-TRAF6 in the absence or presence of different concentrations of Myc-CRBN. At 38 h after transfection, transfected cells were extracted, immunoprecipitated with an anti-Flag antibody, and then immunoblotting was performed with anti-Flag, anti-Myc, or anti-HA antibodies. (**f**) THP-1 cells were infected with a lentivirus containing shRNA targeted to human TRAF6 or control shRNA sequences. Two weeks post-infection, endogenous expression of TRAF6 protein was analyzed in TRAF6^KD^ and control (Ctrl) THP-1 cells. (**g**) TRAF6^KD^ and Ctrl THP-1 cells were transfected with mock, Flag-TRAF6, or HA-CRBN vectors, as indicted, together with pBIIx-luc and Renilla luciferase. Twenty-four hours after transfection, cells were untreated or treated with LPS (200 ng/ml) for 6 h and then analyzed for luciferase activity. Results are expressed as the fold induction in luciferase activity relative to that in untreated cells. All error bars represent S.D. of the mean from triplicate samples. *P<0.05. (**h**) TRAF6^KD^ and Ctrl THP-1 cells were transfected with mock, Flag-TRAF6, or HA-CRBN vectors, as indicated, and treated with or without LPS (200 ng/ml) for 9 h, then, production of IL-6 was analyzed by ELISA. All error bars represent S.D. of the mean from triplicate samples. *P<0.05 and **P<0.01

Having shown that CRBN has no effect on the formation of the TAK1–TAB1–TAB2 complex, we next examined whether CRBN affects the formation of the TRAF6–TAB2 complex. An IP assay revealed that Flag-TRAF6 significantly co-precipitated with HA-CRBN (Figure 4a, lane 3). To identify the interaction domain of TRAF6 in CRBN, truncated TRAF6 mutants were generated (Figure 4b; Supplementary Table 1), and then co-transfected into HEK293T cells along with HA-CRBN. HA-CRBN was co-precipitated with Flag-TRAF6 wt and Flag-TRAF6 110-522 (Figure 4c, lanes 1 and 2), but not with Flag-TRAF6 260-522 or Flag-TRAF6 349-522, suggesting that CRBN interacts with the zinc finger domain of TRAF6. K124 in TRAF6 is required for IL-1-dependent ubiquitination and IKK activation.^24^ As K124 is located in the zinc finger domain of TRAF6, we assessed whether the interaction of CRBN with the zinc finger domain of TRAF6 would affect the ubiquitination of TRAF6. Flag-TRAF6 was co-transfected into HEK293T cells along with Myc-CRBN in the presence or absence of HA-Ub. TRAF6 ubiquitination was quite apparent in the absence of Myc-CRBN, whereas marked attenuation was noted in the presence of Myc-CRBN (Figure 4d, lane 5 *versus* lane 8). To verify the result, Flag-TRAF6 and HA-Ub were co-transfected into HEK293T cells with different concentrations of Myc-CRBN. According to expressions of Myc-CRBN, the ubiquitination of TRAF6 gradually decreased (Figure 4e, lane 2 *versus* lanes 3–5), indicating that CRBN attenuates the ubiquitination of TRAF6.

On the basis of the above results, we examined whether CRBN is negatively involved in the TRAF6-induced activation of NF-*κ*B in response to TLR4 stimulation. To do this, we generated TRAF6-knockdown (TRAF6^KD^) THP-1 cells using a lentivirus containing shRNA targeted to human TRAF6 (Figure 4f). Ctrl or TRAF6^KD^ THP-1 cells were transiently transfected with mock, Flag-TRAF6, and HA-CRBN, as indicted in Figure 4g. As expected, TRAF6^KD^ THP-1 cells displayed decreased NF-*κ*B reporter activity in response to LPS stimulation, as compared with Ctrl cells (Figure 4g, column 2 *versus* column 8). In addition, overexpression of TRAF6 significantly enhanced NF-*κ*B reporter activity in both cells (Figure 4g, column 2 *versus* column 3 in Ctrl; column 8 *versus* column 9 in TRAF6^KD^). Interestingly, TRAF6-induced activation of NF-*κ*B was significantly attenuated by the expression of CRBN in a dose-dependent manner (Figure 4g, column 3 *versus* columns 4, 5, and 6 in Ctrl; column 8 *versus* columns 10, 11, and 12 in TRAF6^KD^). Production of the cytokine IL-6 was also markedly enhanced in both groups of cells transfected with Flag-TRAF6 (Figure 4h, column 2 *versus* column 3 in Ctrl; column 6 *versus* column 7 in in TRAF6^KD^), whereas the effect was significantly suppressed in the cells co-transfected with Flag-TRAF6 and HA-CRBN (Figure 4h, column 3 *versus* column 4 in Ctrl; column 7 *versus* column 8 in in TRAF6^KD^), strongly suggesting that CRBN functionally regulates TRAF6 through attenuation of ubiquitination.

**Figure 5:**
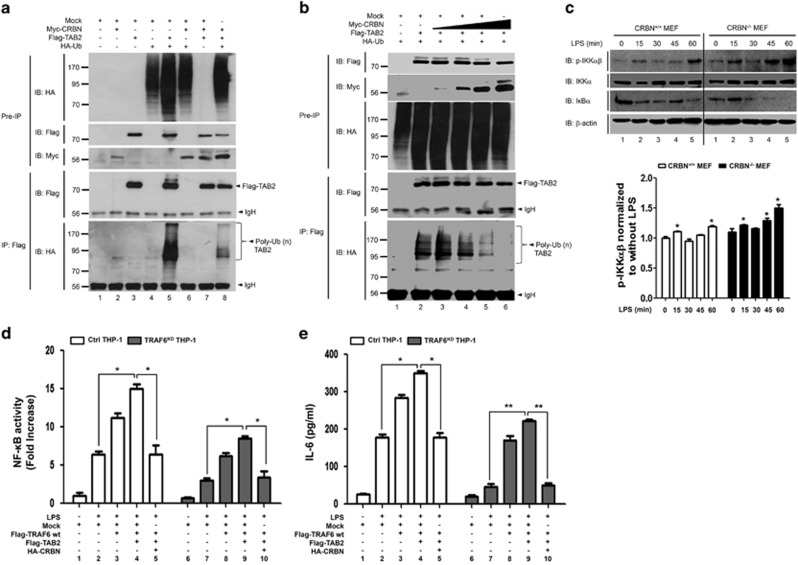
CRBN inhibits ubiquitination of TAB2. (**a**) HEK293T cells were transfected with mock, Myc-CRBN, Flag-TAB2, or HA-Ub, as indicated. At 38 h after transfection, transfected cells were extracted, immunoprecipitated with anti-Flag antibody, and then immunoblotting was performed with anti-Flag, anti-Myc, or anti-HA antibodies. (**b**) HEK293T cells were transfected with mock, HA-Ub, and Flag-TAB2 in the absence or presence of different concentrations of Myc-CRBN. At 38 h after transfection, transfected cells were extracted, immunoprecipitated with an anti-Flag antibody, and then immunoblotting was performed with anti-Flag, anti-Myc, or anti-HA antibodies. (**c**) CRBN^+/+^ or CRBN^*−/−*^ MEF cells were stimulated without or with LPS for different periods of time, as indicated, then subjected to extraction, and western blotting was performed with the antibodies indicated on the left. The band intensity of p-IKK*α*/*β* was analyzed with Image J (bottom). All error bars represent S.D. of the mean from three independent experiments *P<0.05 *versus* without LPS. (**d**) TRAF6^KD^ and Ctrl THP-1 cells were transfected with mock, Flag-TRAF6 wt, Flag-TAB2, or HA-CRBN vectors, as indicted, together with pBIIx-luc and Renilla luciferase. Twenty-four hours after transfection, cells were untreated or treated with LPS (200 ng/ml) for 6 h and then analyzed for luciferase activity. Results are expressed as the fold induction in luciferase activity relative to that in untreated cells. All error bars represent S.D. of the mean from triplicate samples. *P<0.05. (**e**) TRAF6^KD^ and Ctrl THP-1 cells were transfected with mock, Flag-TRAF6 wt, Flag-TAB2, or HA-CRBN vectors, as indicted, and treated with or without LPS (200 ng/ml) for 9 h, and production of IL-6 was analyzed by ELISA. All error bars represent S.D. of the mean from triplicate samples. *P<0.05 and **P<0.01

Ubiquitinated TRAF6 may activate TAK1 through ubiquitination of TAB2.^23, 33^ Therefore, we examined whether the effect of CRBN on TRAF6 ubiquitination leads to the ubiquitination of TAB2. Flag-TAB2 was co-transfected into HEK293T cells along with Myc-CRBN in the presence or absence of HA-Ub. The ubiquitination of TAB2 was significantly apparent in the absence of Myc-CRBN, whereas marked attenuation was evident in the presence of Myc-CRBN (Figure 5a, lane 5 *versus* lane 8). To verify this result, Flag-TAB2 and HA-Ub were co-transfected into HEK293T cells with different concentrations of Myc-CRBN. The ubiquitination of TAB2 was gradually decreased in a Myc-CRBN dose-dependent manner (Figure 5b, lane 2 *versus* lanes 3-6). As ubiquitination of TRAF6 and TAB2 is critically important for the activation of the IKK complex,^23, 33^ we therefore examined whether a deficiency in CRBN positively regulates the activation of IKKs in response to TLR4 stimulation. In order to do that, CRBN^+/+^ or CRBN^*−/−*^ MEF cells were stimulated without or with LPS for different times, as indicated in Figure 5c. Interestingly, phosphorylated IKK*αβ* was significantly elevated in CRBN^*−/−*^ MEF cells in response to LPS stimulation, and that led to the degradation of I*κ*B-*α*, which was not the case in CRBN^+/+^ MEF cells (Figure 5c, CRBN^*−/−*^ *versus* CRBN^+/+^ MEF cells), suggesting that the inhibition of TRAF6 and TAB2 ubiquitination by CRBN is negatively associated with IKK activation. To functionally verify the results, Ctrl or TRAF6^KD^ THP-1 cells were transiently transfected with mock, Flag-TRAF6 wt, Flag-TAB2, and HA-CRBN constructs, as indicted in Figure 5d, and then cells were stimulated with or without LPS. Overexpression of TRAF6 and TAB2 significantly enhanced NF-*κ*B reporter activity in both cell types (Figure 5d, column 2 *versus* column 4 in Ctrl; column 7 *versus* column 7 in TRAF6^KD^). Interestingly, the TRAF6 and TAB2-induced activation of NF-*κ*B was significantly attenuated by expression of CRBN (Figure 5d, column 4 *versus* column 5 in Ctrl; column 9 *versus* column 10 in TRAF6^KD^). In terms of the production of IL-6, similar results were evident in both cell types (Figure 5e), indicating that the negative effect of CRBN on TRAF6 ubiquitination is linked to suppression of TAB2 ubiquitination, which leads to inhibition of NF-*κ*B activation.

### CRBN negatively regulates NF-*κ*B-dependent gene expression and septic shock response to LPS challenge

**Figure 6:**
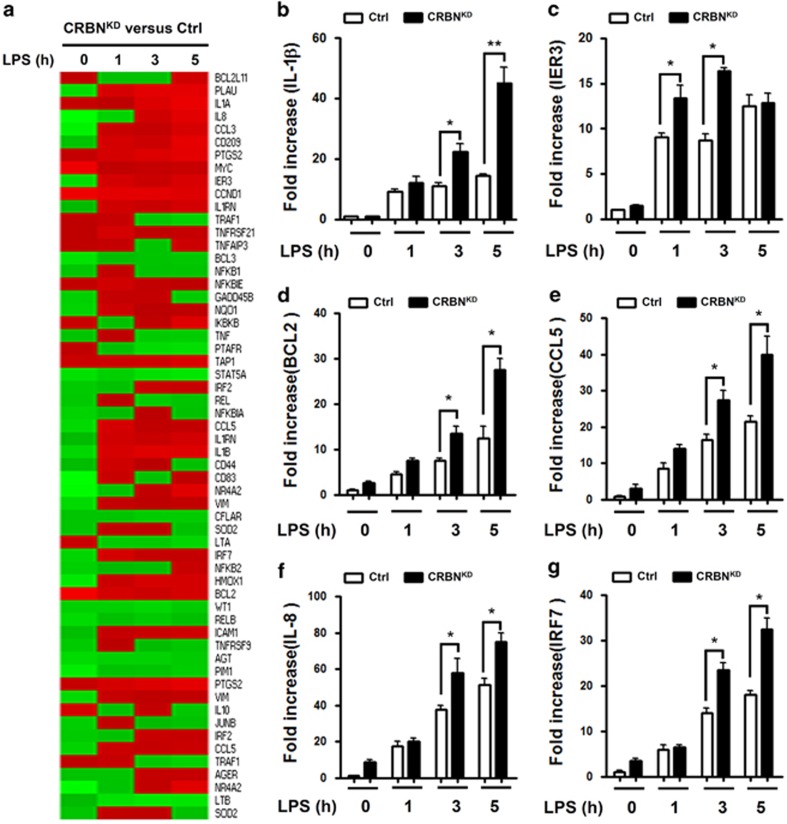
CRBN-knockdown THP-1 cells enhance NF-*κ*B-dependent gene expression. (**a**) Ctrl THP-1 and CRBN^KD^ THP-1 cells were treated with or without LPS (200 ng/ml) for different lengths of time, as indicated. NF-*κ*B-dependent gene expression was compared at each time point (CRBN^KD^ *versus* Ctrl THP-1). (**b**–**g**) RNA was extracted from Ctrl and CRBN^KD^ THP-1 cells treated with or without LPS (200 ng/ml) for different periods of time, as indicated, followed by qRT-PCR analysis with primers specific for the IL-1*β* (**b**), IER3 (**c**), BCL2 (**d)**, CCL5 (**e**), IL-8 (**f**), and IRF7 (**g**) genes. Data represent the average of data from two independent experiments, each conducted in triplicate. Error bars represent the mean±S.D. of six samples. *P<0.05 and **P<0.01

Finally, we examined the functional role of CRBN in *ex vivo* and *in vivo* systems. Microarray analysis was done to assess whether CRBN knockdown in THP-1 cells is functionally able to regulate NF-*κ*B-dependent genes induced by TLR4 stimulation. Following stimulation with LPS, marked changes in gene expression profiles could be detected (Supplementary Figure 1). To assess NF-*κ*B-dependent gene expression with LPS stimulation, NF-*κ*B-dependent genes containing specific *κ*B-binding DNA sequences were further sorted out. Expression levels of genes were significantly altered in Ctrl or CRBN^KD^ THP-1 cells not treated or treated with LPS (Figure 6a). To verify the results, quantitative real-time PCR (qRT-PCR) analysis was done using specific primers targeted to the IL-1*β*, IER3, BCL2, CCL5, IL-8, and IRF7 genes (Figures 6b and g). These genes were significantly upregulated in LPS-treated Ctrl THP-1 cells, as compared with non-stimulated cells (Figures 6b and g, without LPS *versus* with LPS in Ctrl cells). Moreover, gene expression was markedly elevated in CRBN^KD^ THP-1 cells compared with LPS-treated Ctrl THP-1 cells (Figures 6b–g, open bars *versus* closed bars), indicating that CRBN negatively regulates NF-*κ*B-dependent gene expression induced by TLR4 stimulation.

**Figure 7:**
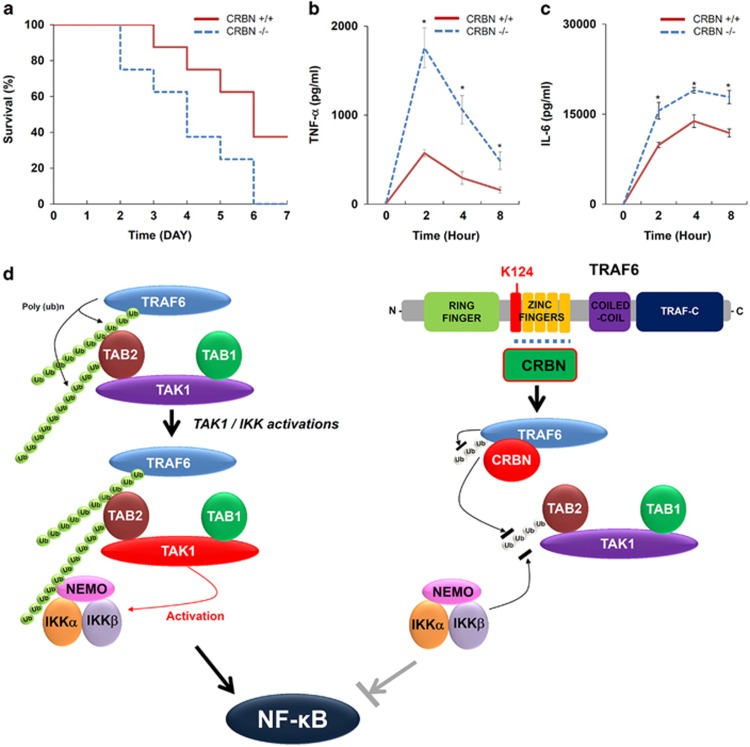
*Crbn*^−/−^ mice exhibit a higher mortality rate following LPS challenge. (**a**) *Crbn*^−/−^ (*n*=8) and *Crbn*^+/+^ (*n*=8) mice were injected intraperitoneally with 7.5 mg/kg LPS in 100 *μ*l of PBS. Survival was monitored for 7 days after LPS challenge. (**b,c**) Serum from mice taken at different times after LPS challenge was isolated, and the concentrations of the TNF-*α* (**b**) and IL-6 (**c**) cytokines were measured with a BD Cytometric Bead Array. Error bars represent the mean±S.D. of eight samples. *P<0.05 (**d**) A schematic model of the negative regulation of CRBN in TLR4 signaling. Upon TLR4 stimulation, ubiquitinated TRAF6 is associated with the TAB2–TAK–TAB1 complex though the interaction between its poly-ubiquitinated chain and TAB2, TAB2 is ubiquitinated, and the complex facilities the activation of TAK1. Simultaneously, the IKK complex is associated with the former complex through the poly-ubiquitinated chain and TAB2, leading to the activation of IKKs (left). In contrast, the interaction between CRBN and TRAF6 may lead to the attenuation of ubiquitination of TRAF6 by masking the K124 residue of TRAF6 and inhibiting the association of the TAK1–TAB1–TAB2 complex to TRAF6, leading to inhibition of ubiquitination of TAB2 and decreased association of the IKK complex with ubiquitinated TAB2 (right)

Next, we examined whether the mortality rate in CRBN-deficient mice (*Crbn*^−/−^) is critically affected by LPS challenge, and whether the effects are associated with increases in levels of pro-inflammatory cytokines. CRBN-deficient (*Crbn*^−/−^) and control mice (*Crbn*^+/+^) were challenged with LPS (7.5 mg/kg intraperitoneally), and then the survival rate was monitored over time. Following LPS challenge, the higher mortality rate in *Crbn*^−/−^ mice was significantly greater than that of *Crbn*^+/+^ mice (Figure 7a, *Crbn*^−/−^ *versus Crbn*^+/+^). Half of the *Crbn*^−/−^ mice died within 4 days of LPS challenge, whereas 90% of *Crbn*^+/+^ mice survived for this period of time (Figure 7a). In addition, 100% mortality of *Crbn*^−/−^ mice occurred within 6 days of LPS challenge, whereas 40% of *Crbn*^+/+^ mice survived for more than 6 days (Figure 7a), indicating that *Crbn*^−/−^ mice are more susceptible to LPS challenge. To examine whether mortality is associated with an enhancement in serum levels of pro-inflammatory cytokines, TNF-*α* and IL-6 levels were measured in response to LPS exposure. The levels of both cytokines were significantly higher in *Crbn*^−/−^ mice than in *Crbn*^+/+^ mice (Figure 7b, TNF-*α*; Figure 7c, IL-6). These results strongly suggest that *Crbn*^−/−^ mice exhibit a higher mortality rate following LPS challenge, and the effects may be critically related to the production of pro-inflammatory cytokines, such as IL-6 and TNF-*α*, which are regulated by NF-*κ*B.

## Discussion

We provide evidence that CRBN negatively regulates TLR4-mediated signaling. Biochemical studies to deduce the molecular mechanism revealed that CRBN interacts with TAK1 and TRAF6, but not TAB1 and TAB2. As the association of TAB1 and TAB2 proteins to TAK1 is critically implicated with TAK1 activation,^25, 26, 27^ we examined whether the interaction of CRBN and TAK1 affects the molecular association of TAB1 and TAB2. We found that CRBN does not interrupt the association between TAB1 and TAB2 to TAK1 (Figure 3h). Interestingly, CRBN interacted with TRAF6 through the zinc finger domain of TRAF6. Regarding the ubiquitination of TRAF6 in TLR4 signaling, the autoubiquitination site (K124) of TRAF6 is located in the zinc finger domain and is functionally critical for the association of the TAK1–TAB2 complex.^24^ According to data on the overexpression of CRBN, ubiquitination of TRAF6 and TAB2 were markedly attenuated, strongly indicating that CRBN-mediated inhibition of TLR4 signaling might be critically associated with the suppression of TRAF6 and TAB2 ubiquitination. We also confirmed the negative role of CRBN in TLR4 signaling by using CRBN^KD^ THP-1 cells and CRBN-knockout mice. CRBN^KD^ THP-1 cells showed enhanced NF-*κ*B activity and production of pro-inflammatory cytokines in response to TLR4 stimulation. As expected, the susceptibility to LPS challenge was significantly increased in the CRBN-knockout mice, accompanying elevated TNF and IL-6 levels, supporting the negative regulation of CRBN in TLR4 signaling.

There is a previously identified functional role of CRBN in the CRL4^CRBN^ E3 ligase.^6, 13, 14^ Within the CRL4^CRBN^ E3 ligase complex, DDB1 functions as the adaptor connecting the CRBN substrate receptor to the ligase. In regard to substrates for the CRL4^CRBN^ E3 ligase, endogenous substrates have been identified.^6, 13, 14^ For the ubiquitination of substrates by CRL4^CRBN^ E3 ligase, the substrate that interacts with CRBN is recruited into the CRL4^CRBN^ E3 ligase through the adaptor DDB1 protein, indicating that CRBN as a substrate receptor has a key role in the ubiquitination process.^13^ Importantly, a recent report has shown that CRBN functions via a ubiquitin-independent chaperone-like mechanism to mediate the folding and maturation of the CD147 and MCT1 proteins, thereby allowing activation of the CD147–MCT1 transmembrane complex.^34^ Interestingly, IMiDs abrogate this mechanism in a competitive manner to mediate their antitumor and teratogenic activities. The result has provided new insights into CRBN function and IMiD biology such as an ubiquitin-independent function for CRBN and as a new mode of action for IMiD. Moreover, CRBN-independent processes make a significant contribution to the anti-inflammatory properties of thalidomide in mice.^35^ They showed that IMiDs are effective inhibitors of TLR4-induced type-1 interferon production via suppression of the TRIF/IRF3 pathway. Nevertheless, the molecular mechanisms by which CRBN or IMiDs mediate the anti-inflammatory effects have remained elusive. We demonstrate that CRBN masks the autoubiquitination site (K124) of TRAF6, and the CRBN–TRAF6 interaction inhibits TRAF6 ubiquitination, thereby resulting in inhibition of the recruitment of TAB2 to TRAF6 and the ubiquitination of TAB2. The inhibitory effect was critically associated with the suppression of NF-*κ*B activity, resulted in attenuations of the production of pro-inflammatory cytokines. Although it cannot be ruled out that IMiDs are implicated in the regulatory mechanism, we speculate that CRBN itself functions as a binding protein to the autoubiquitination site of TRAF6, negatively regulating TLR4-mediated signaling.

In terms of the regulation and control of immune responses, the negative regulators, like CRBN, may be very important. Since excessive or prolonged inflammatory responses to microbial infection can lead to harmful effects on the host, inflammatory signals including TLRs-related signaling need to be tightly controlled in the host.^36^ So far, various mechanisms and cellular proteins capable of interrupting TLRs-mediated signaling have been proposed and reported.^31, 32^ Negative regulation of TLRs signaling occurs in the membrane, cytoplasm, and nucleus through distinct inhibitors affecting multiple signaling steps. As mentioned above, the ubiquitination of TRAF6 critically affects downstream signaling molecules, indicating that TRAF6 ubiquitination is a plausible target for the control of TLRs-related signaling. Interestingly, various mechanisms by which TRAF6 is regulated have been discovered.^37, 38, 39, 40, 41^ A recent report showed that MST4 limits inflammatory responses through direct interaction and phosphorylation of the adaptor protein TRAF6.^42^ They found that modification of TRAF6 by MST4 induced impairments in homo-oligomeric association and thus the assembly of TRAF6-mediated signaling complexes, which in turn impeded K63-linked autoubiquitination of TRAF6 and downstream NF-*κ*B signaling. These results strongly suggest that TRAF6 ubiquitination has a pivotal role in TLR-mediated signaling, thereby providing a potential cellular target for modulating excessive inflammatory responses under certain physiological conditions.

In conclusion, the ubiquitination of TRAF6 has an essential role for the association of the TAK1–TAB1–TAB2 complex via the interaction between the poly-ubiquitination chain of TRAF6 and TAB2 (Figure 7d, left). The association in turn leads to the ubiquitination of TAB2 and facilitates the activation of TAK1 and IKK complex. However, the interaction between CRBN and TRAF6 may lead to the attenuation of ubiquitination of TRAF6 by masking the K124 residue of TRAF6 and inhibit the association of the TAK1–TAB1–TAB2 complex to TRAF6, leading to inhibition of ubiquitination of TAB2 and the association of the IKK complex with ubiquitinated TAB2 (Figure 7d, right).

## Materials and Methods

### Mice and animal experiments

CRBN-deficient mice (*Crbn*^−/−^) and control mice (*Crbn*^+/+^) were used as described previously.^43^ For LPS challenge, *Crbn*^−/−^ (*n*=8) and *Crbn*^+/+^ (*n*=8) mice were injected intraperitoneally with 7.5 mg/kg LPS (Sigma-Aldrich, St. Louis, MO, USA) in 100 *μ*l of phosphate buffered saline (PBS). Survival was monitored for 7 days after LPS challenge. To measure cytokines levels, sera was obtained from mice at different times after LPS challenge and the concentrations of the TNF-*α* and IL-6 cytokines were detected by BD Cytometric Bead Array (BD Biosciences, San Jose, CA, USA) according to the manufacturer's recommendations. All animal studies and protocols were approved by the Institutional Animal Use and Care Committee of Gwangju Institute of Science and Technology.

### Cells and antibodies

HEK293 cells expressing human TLR4 (293/TLR4) were purchased from InvivoGen (San Diego, CA, USA) and were maintained according to the manufacturer's protocol. HEK293T human embryonic kidney cells were purchased from the American Type Culture Collection (ATCC, Manassas, VA, USA) and were maintained in Dulbecco's modified Eagle's medium (DMEM; Invitrogen, Carlsbad, CA, USA) containing 10% fetal bovine serum (FBS), 2 mM  l-glutamine, 100 units/ml penicillin, 100 *μ*g/ml streptomycin, and 5 × 10^−5^ M *β*-mercaptoethanol. CRBN^+/+^ and CRBN^−/−^ MEFs were isolated from E14.5 embryos born to heterozygous intercrosses, cultured in DMEM (Life Technologies, Carlsbad, CA, USA) with 10% (v/v) FBS (Hyclone), and assayed at passages 3–6. THP-1 human monocytic cells were purchased from ATCC and were maintained in RPMI medium (Invitrogen) containing 10% FBS, 2 mM  l-glutamine, 100 units/ml penicillin, 100 *μ*g/ml streptomycin, and 5 × 10^−5^ M *β*-mercaptoethanol. Lentivirus containing small hairpin RNA (shRNA) targeting human CRBN (sc-78528-V), lentivirus containing shRNA targeting human TRAF6 (sc-36717-V), or control shRNA lentivirus (sc-108080) were purchased from Santa Cruz Biotechnology (Santa Cruz, CA, USA). THP-1 cells were cultured in wells of a 24-well plate (2 × 10^5^ cells per well) and infected with lentivirus according to the manufacturer's protocol. Ctrl THP-1 cells, CRBN-knockdown (CRBN^KD^) THP-1 cells, and TRAF6-knockdown (TRAF6^KD^) THP-1 cells were selected in puromycin-containing (4–8 *μ*g/ml) medium and cultured as described previously.^44, 45^ Specific anti-HA, anti-Flag, anti-Myc, anti-TRAF6, and anti-GAPDH antibodies were from Cell Signaling Technology (Beverley, MA, USA). The anti-CRBN antibody was from Abcam (Cambridge, MA, USA). Mouse TrueBlot ULTRA: Anti-Mouse Ig HRP was from Rockland Immunochemicals Inc. (Limerick, PA, USA).

### Luciferases reporter assay

293/TLR4 cells, Ctrl THP-1, CRBN^KD^ THP-1 cells, or TRAF6^KD^ THP-1 cells were transiently transfected with different vectors, as indicated in the figures, using Lipofectamine LRX (Invitrogen) or the Neon transfection system (Invitrogen), together with the pBIIx-luc NF-*κ*B-dependent reporter construct and the Renilla luciferase vector (Promega, Madison, WI, USA). At 24 h posttransfection, the cells were treated or not with LPS (200 ng/ml) for 6 h and lysed, and luciferase activity was measured using a dual luciferase assay kit (Promega).

### Measurement of pro-inflammatory cytokines and p65- and p50-DNA-binding assays

Wt THP-1, Ctrl THP-1, or CRBN^KD^ THP-1 cells were untreated or treated with LPS (200 ng/ml) for 9 h and the supernatants were collected. The levels of human IL-1*β* and IL-6 were measured in the supernatants according to the manufacturer's protocol (R&D Systems, Minneapolis, MN, USA). Ctrl THP-1 and TRAF6^KD^ THP-1 cells were transfected with mock, Flag-TRAF6 wt, Flag-TAB2, and/or HA-CRBN, as indicated in each figure. Cell were untreated or treated with LPS (200 ng/ml) for 9 h and the supernatants were collected. The level of human IL-6 was measured in the supernatants according to the manufacturer's protocol (R&D Systems). For p65- or p50-DNA-binding assay, cells were transiently transfected with mock or HA-CRBN vector. After 38 h, nuclear proteins from transfectants treated for 6 h with or without LPS (200 ng/ml) were prepared with the CelLytic NuCLEAR extraction kit in accordance with the manufacturer's protocol (Sigma-Aldrich). Activities of the transcription factors p65 and p50 were determined with the TransAM NF-*κ*B transcription factor assay kit according to the manufacturer's instructions (Active Motif North America, Carlsbad, CA, USA).

### Plasmids

HA-tagged CRBN, Flag-tagged CRBN, Myc-tagged CRBN, Myc-tagged TAK1, Flag-tagged TAK1, Flag-tagged TRAF6, Flag-tagged TAB1, Flag-tagged TAB2, and HA-tagged Ub vectors were used. Myc-tagged TAK1 truncation mutants were generated as described previously.^43^ To generate Flag-tagged TRAF6 truncated mutants, specific primers were designed as described in Supplementary Table 1, and each truncated mutant was generated by PCR.

### Western blotting and immunoprecipitation (IP) assay

Cells were transfected with the appropriate vectors, as indicated in each figure. Western blotting and IP assays were performed as described previously.^46, 47, 48, 49^ The detailed procedures for the IP assay are described in Supplementary Materials and Methods. CRBN^+/+^ or CRBN^*−/−*^ MEF cells were stimulated without or with LPS for different times, lysed in lysis buffer, and the lysates were examined by western blotting with anti-pho-IKK*αβ* (Cell Signaling Technology), anti-IKK*α* (Cell Signaling Technology), anti-I*κ*B-*α* (Cell Signaling Technology), and anti-*β*-actin (Cell Signaling Technology) antibodies.

### Ubiquitination assay

HEK293T cells were transiently transfected with Myc-CRBN, Flag-TRAF6, or Flag-TAB2, as indicated in the figures, along with an HA-Ub vector. At 36 h after transfection, transfected cells were extracted and immunoprecipitated with an anti-Flag antibody. Immunoprecipitated complexes were separated by 6–10% SDS-PAGE and probed with anti-HA or anti-Flag antibody.

### Microarray analysis

Microarray analysis, raw data preparation, and statistical analysis were performed as described in previous reports.^45, 48, 50, 51^ Briefly, Ctrl and CRBN^KD^ THP-1 cells were treated with or without LPS (200 ng/ml) for different times. Total RNA was isolated, and RNA purity and integrity were evaluated with an ND-1000 Spectrophotometer (NanoDrop Technologies, Wilmington, DE, USA) and an Agilent 2100 Bioanalyzer (Agilent Technologies, Palo Alto, CA, USA). Total RNA was amplified and purified using TargetAmp-Nano Labeling Kit for Illumina Expression BeadChip (EPICENTRE, Madison, WI, USA) to yield biotinylated cRNA according to the manufacturer's instructions. For the hybridization experiment, 750 ng of labeled cRNA samples were hybridized to each Human HT-12 v4.0 Expression Beadchip for 17 h at 58 °C, according to the manufacturer's instructions (Illumina, Inc., San Diego, CA, USA). Detection of array signal was carried out using fluorolink streptavidin-Cy3 (GE Healthcare Biosciences, Little Chalfont, UK) following the bead array manual. For raw data preparation and statistic analysis, the quality of hybridization and overall chip performance were monitored by visual inspection of both internal quality control checks and the raw scanned data. Raw data were extracted using the software provided by the manufacturer (Illumina GenomeStudio v2011.1 (Gene Expression Module v1.9.0); Illumina, San Diego, CA, USA)). Statistical significance of the expression data was determined using fold change. For a DEG set, hierarchical cluster analysis was performed using complete linkage and Euclidean distance as a measure of similarity. Gene-Enrichment and Functional Annotation analysis for a significant probe list was performed using DAVID (http://david.abcc.ncifcrf.gov/home.jsp). All data analysis and visualization of differentially expressed genes was conducted using R 3.0.2 (www.r-project.org).

### Quantitative real-time polymerase chain reaction (qRT-PCR)

Ctrl and CRBN^KD^ THP-1 cells were treated with or without LPS (200 ng/ml) for different periods of time. Total RNA was isolated and cDNA was synthesized following the manufacturer's protocol (Qiagen, Erkrath, Germany). Primers (all from Qiagen) were: hIL-8 (PPH00568A-200), hIRF7 (PPH02014E-200), hCCL5 (PPH00703B-200), hBCL2 (PPH00079B-200), hIER3 (PPH10008F-200), and hIL-1*β* (PPH00171C-200). qRT-PCR analysis was performed using Rotor-Gene Q (Qiagen) according to the manufacturer's protocol.

### Statistical analysis

*In vitro* data are presented as mean±S.D. of the mean from triplicate samples. Comparisons were statistically assessed using the Student's *t*-test. *P* values <0.05 (or <0.01, as indicated) were considered to be statistically significant.

## Abbreviations

CRBN: Cereblon
TLRs: toll-like receptors
TNF: tumor necrosis factor
TRAF6: TNF receptor associated factor 6
NF-*κ*B: nuclear factor-kappa B
TAK1: transforming growth factor-beta (TGF-*β*)-activated kinase 1
TAB: TAK1-binding protein
LPS: lipopolysaccharide
AMPK: AMP-activated protein kinase
CRBN^KD^: CRBN-knockdown
TRAF6^KD^: TRAF6-knockdown
Ctrl: control
Wt: wild type

## Supplementary Material

Supplementary FInformation
