# Supplementary material for: Cereblon negatively regulates TLR4 signaling through the attenuation of ubiquitination of TRAF6
Source: Cell Death Dis. 2016 Jul 28;7(7):e2313–. doi: 10.1038/cddis.2016.226 (PMC4973362; doi:10.1038/cddis.2016.226)
Supplement: Supplementary FInformation [file cddis2016226x1.doc]

**Supplementary Materials and Methods**

**Immunoprecipitation (IP) assay**. HEK293T cells were transfected with mock and HA-CRBN, mock and Flag-TAK1, HA-CRBN and Flag-TAK1, or HA-CRBN and Flag-TRAF6. At 38 h after transfection, transfected cells were extracted and immunoprecipitated with anti-Flag antibody. The interaction was detected by western blotting with anti-Flag or anti-HA antibody. HEK293T cells were transfected with mock and HA-CRBN, mock and Flag-TAK1, mock and Flag-TAB1, mock and Flag-TAB2, HA-CRBN and Flag-TAK1, HA-CRBN and Flag-TAB1, or HA-CRBN and Flag-TAB2, respectively. At 38 h after transfection, transfected cells were extracted and immunoprecipitated with anti-Flag or HA antibody. The interaction was detected by western blotting with anti-Flag or anti-HA antibody. For the domain mapping of TAK1 to interact with CRBN, HEK293T cells were transfected with Myc-TAK1 wild type (wt) or Myc-TAK1 1-500, Myc-TAK1 1-400, Myc-TAK1 1-300, Myc-TAK1 1-200, or Myc-TAK1 1-100, along with Flag-CRBN. At 38 h after transfection, transfected cells were extracted and immunoprecipitated with anti-Myc antibody. The interaction was detected by Western blotting with anti-Myc or anti-Flag antibody. To identify the interaction domain of TAK1 to TAB1 or TAB2, HEK293T cells were transfected with Myc-TAK1 wt or Myc-TAK1 1-500, Myc-TAK1 1-400, or Myc-TAK1 1-300, along with Flag-TAB1 or Flag-TAB2. At 38 h after transfection, transfected cells were extracted and immunoprecipitated with anti-Myc antibody. The interaction was detected by Western blotting with anti-Myc or anti-Flag antibody. For the competitive interaction, HEK293T cells were transfected with Myc-TAK1 and Flag-TAB1 or Myc-TAK1 and Flag-TAB2 in the presence or absence of different concentrations of HA-CRBN, respectively. At 38 h after transfection, transfected cells were extracted and immunoprecipitated with anti-Myc antibody. The interaction was detected by Western blotting with anti-Myc, anti-HA, or anti-Flag antibody. For the domain mapping of TRAF6 to interact with CRBN, HEK293T cells were transfected with HA-CRBN and Flag-TRAF6 wt, Flag-TRAF6 110-522, Flag-TRAF6 260-522, or Flag-TRAF6 349-522. At 38 h after transfection, transfected cells were extracted and immunoprecipitated with anti-Flag antibody. The interaction was detected by western blotting with anti-HA or anti-Flag antibody.
