# Supplementary material for: Cereblon negatively regulates TLR4 signaling through the attenuation of ubiquitination of TRAF6
Source: Cell Death Dis. 2016 Jul 28;7(7):e2313–. doi: 10.1038/cddis.2016.226 (PMC4973362; doi:10.1038/cddis.2016.226)
Supplement: Supplementary Table 1 [file cddis2016226x2.doc]

**Supplementary Table 1.** Specific primer pairs used in the construct of TRAF6-truncated mutants

**Constructs Sequences (5’- 3’)**

TRAF6 110-522 Forward GCG AAGCTTATGGAA ATACTGCTGGAA AAT CAA CT

Reverse AACTCGAGCTATACCCCTGCATCAGTACT

TRAF6 260-522 Forward ATAAGCTTATGCGCCACCTACAAGAGA ACA

Reverse AACTCGAGCTAT ACCCCTGCATCAGTACT

TRAF6 349-522 Forward GCGCAAGCT TAT GTGCAATGGAAT TTATATTTGGAAG

Reverse AACTCGAGCTATACCCCTGCA TCAGTACT
