# Supplementary figures and images for: Cereblon negatively regulates TLR4 signaling through the attenuation of ubiquitination of TRAF6
Source: Cell Death Dis. 2016 Jul 28;7(7):e2313–. doi: 10.1038/cddis.2016.226 (PMC4973362; doi:10.1038/cddis.2016.226)

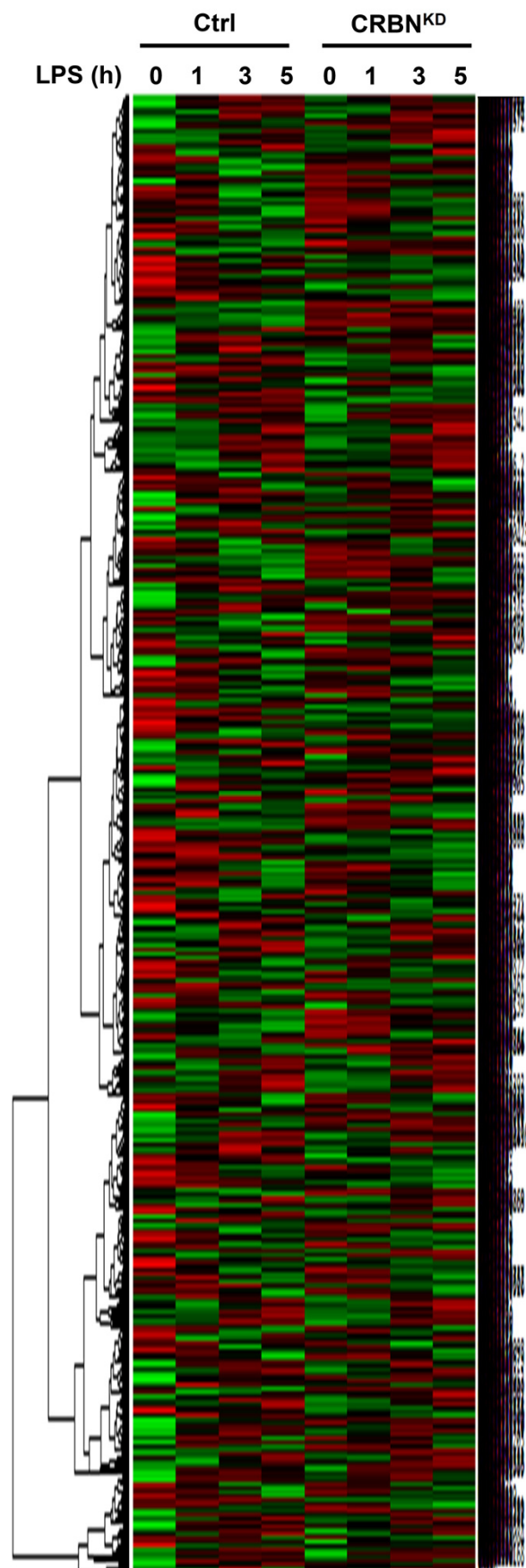

Supplement: Supplementary Figure 1 [file cddis2016226x3.pdf]
