# Supplementary material for: Cereblon negatively regulates TLR4 signaling through the attenuation of ubiquitination of TRAF6
Source: Cell Death Dis. 2016 Jul 28;7(7):e2313–. doi: 10.1038/cddis.2016.226 (PMC4973362; doi:10.1038/cddis.2016.226)
Supplement: Supplementary Figure 1 Legend [file cddis2016226x4.doc]

**Supplementary Figure Legends**

**Supplementary Figure 1:** Comparison of gene expressions in control (Ctrl) THP-1 cells and CRBN^KD^ THP-1 cells. Ctrl and CRBN^KD^ THP-1 cells were treated with or without LPS (200 ng/ ml) for different times, as indicated. RNA was extracted, and then microarray analysis was performed as described in Materials and Methods.
